# The Prognostic Value of the Advanced Lung Cancer Inflammation Index for Major Cardiovascular and Cerebrovascular Events in Patients with Non-ST Elevation Myocardial Infarction Undergoing Percutaneous Coronary Intervention

**DOI:** 10.3390/jcm14051403

**Published:** 2025-02-20

**Authors:** Mehmet Karaca, Muhsin Kalyoncuoğlu, Ahmet Zengin, Semih Eren, Kıvanç Keskin, Ersan Oflar, Mehmet Baran Karataş, Ali Nazmi Çalık

**Affiliations:** 1 Cardiology Department, Atasehir Memorial Hospital, Uskudar University, Istanbul 34662, Turkey; mehmetkaraca06@gmail.com; 2 Bakırköy Sadi Konuk Training and Research Hospital, University of Health Sciences, Istanbul 34758, Turkey; ersanoflar@gmail.com; 3 Dr. Siyami Ersek Thoracic and Cardiovascular Surgery Education Research Hospital, University of Health Sciences, Istanbul 34668, Turkey; ahmetzengin85@gmail.com (A.Z.); semiheren92@hotmail.com (S.E.); kivanckeskin@outlook.com (K.K.); karatasbaran@gmail.com (M.B.K.); calik_nazmi@hotmail.com (A.N.Ç.)

**Keywords:** ALI, MACCE, non-ST elevation myocardial infarction

## Abstract

**Objectives:** Our aim was to investigate whether admission advanced lung cancer inflammation index (ALI) values have a prognostic role on one-year major adverse cardiovascular and cerebrovascular events (MACCEs) in non-ST elevation myocardial infarction (NSTEMI) patients undergoing percutaneous coronary intervention (PCI). **Methods:** Our study consisted of 1173 consecutive patients aged 61.9 ± 12.5 years. The study population was divided into two groups according to the occurrence of MACCEs. BMI (body mass index), serum albumin levels and NLR (neutrophil to lymphocyte ratio) of patients were collected from hospital records, and ALI was calculated based on the following formula: BMI × serum albumin/NLR. We also calculated neutrophil to lymphocyte ratio (NLR), C-reactive protein/albumin ratio (CAR) and uric acid to albumin ratio (UAR) and investigated the association of these inflammation-based biomarkers with one-year MACCEs. **Results:** During the 12-month follow-up period, 158 (13.5%) patients had MACCEs, 55 (4.7%) of whom had all-cause mortality, 96 (8.2%) had nonfatal MI and 7 (0.6%) had nonfatal stroke. Patients with MACCEs had significantly lower ALI (*p* < 0.001), and also ALI (area under the curve [AUC] = 0.658, *p* < 0.001) had better discriminatory power and predictive accuracy in determining one-year MACCEs compared to albumin (AUC = 0.594, *p* < 0.001), NLR (AUC = 0.631, *p* < 0.001), CAR (AUC = 0.595, *p* < 0.001) and UAR (AUC = 0.577, *p* = 0.001) in the ROC analysis. Individuals with an ALI value lower than 43.9 were at greater risk of developing MACCEs (*p* < 0.001) due to the Delong test. **Conclusions:** Determining the level of ALI may have the potential to improve risk prognostication in NSTEMI patients undergoing revascularization therapy.

## 1. Introduction

Non-ST elevation myocardial infarction (NSTEMI) is the most common form of acute coronary syndrome (ACS), accounting for roughly two-thirds of cases, and remains the leading cause of death worldwide [1,2]. Despite significant advances in interventional and medical treatment approaches, long-term outcomes of NSTEMI patients have not improved as seen in ST elevation myocardial infarction (STEMI) [3,4], and these patients continue to experience high rates of mortality and recurrence [5]. Therefore, the identification of patients who are prone to adverse events via detecting modifiable clinical/biochemical risk factors and implementing timely interventions are mandatory to improve overall prognosis [6]. Due to this, recent research has focused on the usefulness of various inflammatory indices in order to assess prognostic risk in ACS patients [7] such as neutrophil to lymphocyte ratio (NLR), C-reactive protein to albumin ratio (CAR) and uric acid to albumin ratio (UAR) [8,9,10,11,12].

On the other hand, malnutrition has been associated with the development of atherosclerosis and an increased incidence of all-cause mortality and major adverse cardiac events in the ACS population [8,13,14]. In this context, ALI, a recently defined nutritional/inflammatory indicator, encompasses both nutritional and inflammatory components, including body mass index (BMI), serum albumin concentration and NLR [8]. The role of ALI in predicting major adverse cardiovascular events in various cardiovascular diseases has recently been established, despite it initially being developed to assess the prognosis of patients diagnosed with non-small cell lung cancer [8,15,16,17,18].

In this perspective, we aimed to investigate the prognostic importance of ALI on long-term follow-up of NSTEMI patients in comparison with CAR, UAR and NLR, which have previously been proven to have predictive roles in this population.

## 2. Materials and Methods

**Figure 1:**
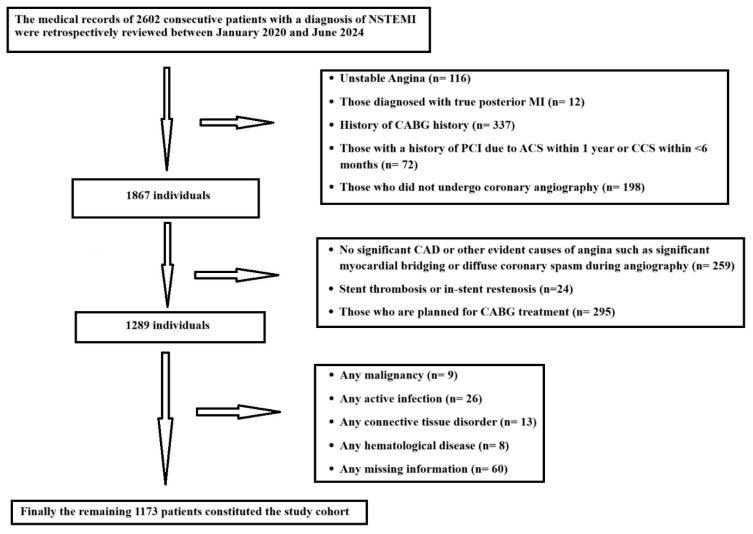
Flow chart of participant selection process and exclusion criteria.

We reviewed the medical records of consecutive patients from January 2020 to December 2024 who were admitted at the University of Health Sciences Department of Cardiology, Dr. Siyami Ersek Thoracic and Cardiovascular Surgery Education Research Hospital, Turkey. Patients who were diagnosed with NSTEMI and underwent coronary angiography with subsequent percutaneous coronary intervention (PCI) during index hospitalization were included in the current study. The participant selection process and exclusion criteria are presented in detail in the study flow chart in Figure 1.

The study cohort consisted of 1173 patients. Clinical and demographic history, including body mass index (BMI), hypertension (HT), diabetes (DM), family history, hyperlipidemia, current smoking status and vascular diseases—defined as previous myocardial infarction (MI), chronic coronary syndrome, PCI and peripheral artery disease (PAD)—were obtained through reviewing the electronic medical records of patients.

The diagnosis of NSTEMI was made according to symptoms, electrocardiographic findings and cardiac enzymes in line with the European Society of Cardiology and American College of Cardiology/American Heart Association guidelines [19,20], and all patients were treated in accordance with these guidelines. Presentation with acute chest pain or overwhelming shortness of breath with the absence of persistent ST elevation is suggestive of non-ST-elevated ACS (except in patients with true posterior myocardial infarction). Non-ST-elevated ACS can be further subdivided based on cardiac biomarkers of necrosis such as cardiac troponin. If cardiac biomarkers are elevated and the clinical findings are appropriate, the patient is considered to have NSTEMI; otherwise, the patient is deemed to have unstable angina pectoris [19,20]. In addition, to identify the patient’s risk, the Global Registry for Acute Coronary Events risk score (GRS) was also calculated, and individuals in all risk groups determined by GRS were included in the study [21].

Patients with hypotension (systolic blood pressure < 90 mmHg or mean arterial pressure < 70 mmHg) combined with tachycardia (heart rate ≥ 100 bpm) associated with symptoms of inadequate vital organ perfusion, such as decreased urine output and altered mental status, were considered hemodynamically unstable [22]. Routine complete blood cell counts and biochemical parameters were obtained at admission and evaluated using an automated blood cell counter and automated chemistry analyzer, respectively.

ALI was calculated according to the patient’s BMI (kg/m^2^), serum albumin level (g/dL) and neutrophil to lymphocyte ratio (NLR), as ALI = BMI × serum albumin/NLR. NLR was the ratio of neutrophil count (10^9^/L) to lymphocyte count (10^9^/L). The CAR was calculated as the ratio of CRP (mg/dL) to the serum albumin (g/dL) concentration. The UAR was calculated as serum uric acid (mg/dL)/serum albumin (g/dL). The study population was divided into two groups as low-ALI (<43.9) and high-ALI (≥43.9) groups according to the cut-off value determined by Youden’s index.

The angiographic images were evaluated by two experienced interventionalists who were blinded to our study. Syntax Score I (SxSI) was used to define the anatomic severity of coronary stenosis (https://syntaxscore.org).

The primary endpoint of our study was MACCEs, which were defined as all-cause mortality, nonfatal myocardial infarction and nonfatal cerebrovascular accident (ischemic stroke or transient ischemic attack).

As our study was retrospectively designed, written informed consent from participants could not be obtained; however, our study protocol was approved by the ethics committee of the University of Health Sciences, Dr. Siyami Ersek Thoracic and Cardiovascular Surgery Education Research Hospital (protocol number: E-28001928-604.01-263774551).

## 3. Statistical Analysis

Normally distributed continuous variables were reported as mean ± standard deviation, and non-normally distributed variables were reported as medians with interquartile ranges. Categorical variables were reported as numbers and percentages. The chi-squared (χ^2^) test was used to compare the categorical variables between the groups. The Kolmogorov–Smirnov test was used to assess whether the variables were normally distributed. The Student’s t-test or the Mann–Whitney U test was used to compare the continuous variables between the groups, according to whether they were normally distributed or not. In order to determine the independent associates, variables associated with the development of MACCEs according to univariate analysis were put in the multivariate Cox regression analysis, with the results reported as hazard ratios (HRs) and 95% confidence intervals (CIs).

Multicollinearity among the independent variables was assessed using the variance inflation factor (VIF) with a cut-off value of <5 to maintain variables in the models. To identify possible multicollinearity among the independent variables, the variance inflation factor (VIF) was calculated using a cut-off value of <5 to maintain variables in the model. To avoid overfitting, we ensured that the “events per variable” rate was at least 10 in our multivariable logistic regression model and also included in the multivariate analysis only variables with higher statistical significance with a *p* value < 0.05 in the univariate analysis. To test the predictive accuracy and capacity to discriminate of the ALI, albumin, NLR, CAR and UAR in determining the MACCEs, the receiving operating characteristic (ROC) curve and the C-statistic [the area under the receiver operating curve], accompanied by a 95% confidence interval, was performed. To compare the discriminatory abilities of the ALI, albumin, NLR, CAR and UAR in determining the development of one-year MACCEs, pairwise comparisons of the ROC curves were performed using the method of DeLong et al. [23]. The optimal cut-off value of ALI was also calculated from the point of maximal sensitivity and specificity using Youden’s index (sensitivity + specificity − 1) [24]. Time to event data were presented graphically with Kaplan–Meier survival curves and long-rank tests. The threshold of statistical significance was established at *p* < 0.05. All statistical analyses were performed using the Statistical Package for the Social Sciences version 24.0 software program (IBM Corp., Armonk, NY, USA). ROC curves of the models were compared using the MEDCALC software program (v.22) (MedCalc Software bv, Ostend, Belgium).

## 4. Results

### 4.1. Baseline Characteristics

A total of 1173 patients (female n = 296, 25.2%) diagnosed with NSTEMI with a mean age of 61.9 ± 12.5 years were included in this study. In terms of clinical and demographic characteristics, while individuals with low ALI had lower BMI (26.9 ± 3.4 vs. 28.4 ± 3.3; *p* < 0.001), lower LVEF (50.3 ± 10.4% vs. 52.2 ± 9.3%; *p* = 0.002), higher GRACE risk score (101.9 ± 28.2 vs. 98.0 ± 25.7; *p* = 0.014) and more hemodynamic instability (4.3% vs. 1.7%, *p* = 0.007) than those with high ALI values, the remaining demographic and clinical parameters were similar (for all *p* > 0.05). Regarding clinical outcome parameters, participants with lower ALI suffered more MACCEs (19.5% vs. 7.7%, *p* < 0.001) except nonfatal stroke (0.5% vs. 0.7%, *p* = 0.744) and were mainly driven by nonfatal MI (12% vs. 4.5%, *p* < 0.001) and all-cause mortality (7% vs. 2.5%, *p* < 0.001).

Additionally, the complexity of coronary artery disease, as defined by the anatomical SxSI, was greater in patients with low ALI (20.7 ± 6.3 vs. 19.6 ± 5.7; *p* = 0.001). Besides that, there was no difference between the groups in terms of postprocedural TIMI < 3 flow (*p* = 0.534), a lack of complete revascularization (*p* = 0.522), suffering from stent thrombosis (*p* = 0.250) and requiring target vessel revascularization (*p* = 0.112).

Considering biochemical and hematological parameters, low-ALI patients had lower serum albumin (3.75 ± 0.47 vs. 4.0 ± 0.44, <0.001), lower hemoglobin (12.9 ± 2.1 vs. 13.9 ± 1.8, *p* <0.001) and fewer lymphocyte counts (median 1.69 vs. median 2.43, *p* < 0.001) in contrast to higher baseline troponin I (median 0.060 vs. median 0.058, *p* = 0.011), higher white blood counts (8.96 ± 2.6 vs. 8.18 ± 1.8, *p* < 0.001) and higher neutrophil counts (6.07 ± 2.1 vs. 4.54 ± 1.2, *p* < 0.001) compared with the high-ALI group. In terms of inflammation-based scoring systems, patients with low ALI had higher NLR (median 3.30 vs. median 1.87, *p* < 0.001), CAR (median 1.51 vs. median 1.30, *p* < 0.001) and UAR (1.54 ± 0.6 vs. 1.37 ± 0.5, *p* < 0.001) values and lower ALI (median 30.8 vs. median 57.9, *p* < 0.001). Detailed demographics, clinical features, laboratory parameters and comparison of the two groups are summarized in Table 1 and Table 2.

### 4.2. Parameters Associated with MACCEs

During twelve months of follow-up, 158 (13.5%) patients developed MACCEs, of whom 55 (4.7%) patients had all-cause mortality, 96 (8.2%) had nonfatal MI and 7 (0.6%) had stroke.

To identify independent predictors of MACCEs, we performed multivariate Cox regression analysis via including variables shown to be significantly associated with MACCEs in univariate analysis (Table 3). Since albumin is a common parameter of the inflammation-based scores, ALI, CAR and UAR, three separate analysis models were performed. and albumin was not included in these models. Moreover, CRP and BMI were not included in any models because they were not found to be associated with MACCEs. Also, since NLR is a member of ALI and uric acid is a member of UAR, it was not included in Model 1 and Model 3, respectively, considering that it would negatively affect the analysis results. In all three analysis models, diabetes, low EF, high SxSI, low eGFR level, high baseline troponin level and a lack of complete revascularization at index hospitalization were found to be independent predictors of one-year MACCEs. When considering inflammatory indicators, NLR (Model 2 and Model 3), ALI (Model 1), CAR (Model 2) and UAR (Model 3) independently related with the development of MACCEs (Table 4).

**Figure 2:**
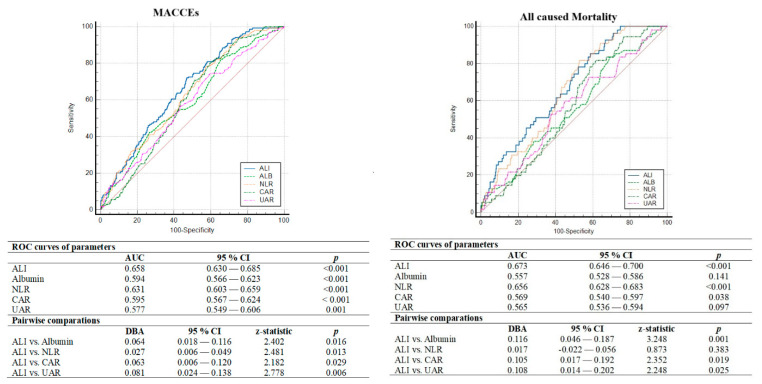
Comparison of predictive performances of ALI, albumin, NLR, CAR and UAR determined by ROC curves in predicting one-year MACCEs and all-cause mortality. Abbreviations: ALI, advanced lung cancer inflammation index; CAR, C-reactive protein to albumin ratio; MACCEs, major adverse cardiovascular and cerebrovascular events; NLR, neutrophil to lymphocyte ratio; ROC, receiver operating characteristic; UAR, uric acid to albumin ratio.

**Figure 3:**
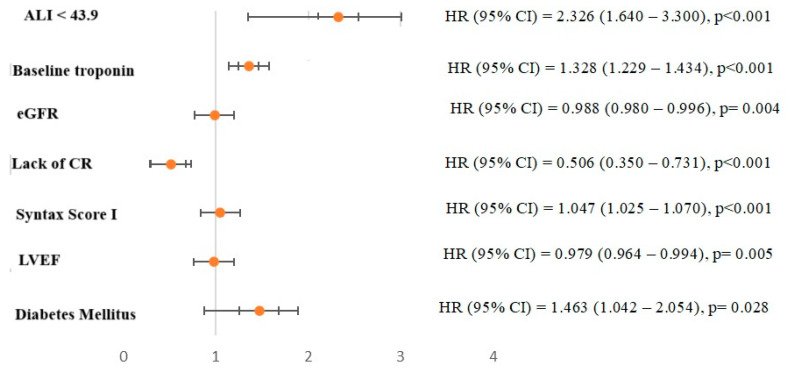
Forrest plot representing hazard ratio of multivariable model Cox regression analysis adjusted for ALI with cut-off value < 43.9. Abbreviations: ALI, advanced lung cancer inflammation index; CI, confidence interval; CR, complete revascularization; eGFR, estimated glomerular filtration rate; HR, hazard ratio; LVEF, left ventricular ejection fraction.

**Figure 4:**
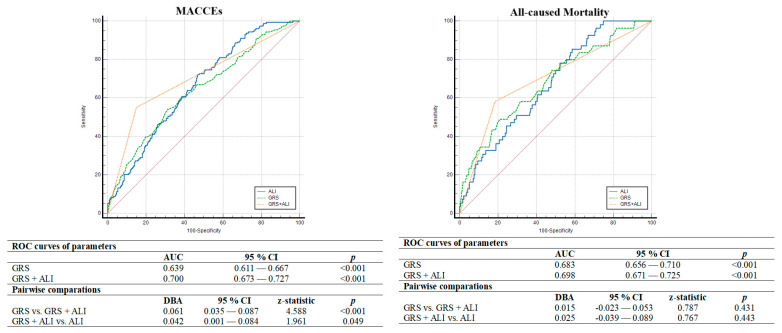
A comparison of the discriminatory ability of GRACE risk score with and without ALI in determining the one-year MACCEs and all-cause mortality. Abbreviations: ALI, advanced lung cancer inflammation index; GRS, GRACE risk score; MACCEs, major adverse cardiovascular and cerebrovascular events.

ROC curve analysis also showed that ALI (AUC = 0.658, *p* < 0.001) had better discriminatory power and association in determining one-year MACCEs compared to albumin (AUC = 0.594, *p* < 0.001), NLR (AUC = 0.631, *p* < 0.001), CAR (AUC = 0.595, *p* < 0.001) and UAR (AUC = 0.577, *p* = 0.001). Moreover, in the comparative analysis of ROC curves using the DeLong test, the discriminative ability of ALI was significantly superior to other inflammatory indices (Figure 2). In addition, ALI less than 43.9 was determined as the cut-off value by using the Youden index with 72% sensitivity and 53% specificity for anticipating the one-year MACCEs. Thus, in multivariate Cox regression analysis considering the established cut-off value, we observed that individuals with ALI < 43.9 face a 2.3-fold increased risk of MACCEs (Figure 3). Additionally, in terms of all-cause mortality, ALI less than 42.75 was determined as the cut-off value with 67% sensitivity and 52% specificity for anticipating the one-year all-cause mortality. Moreover, the DeLong test revealed that ALI had better discrimination power than other inflammatory parameters except for NLR (Figure 2). We also tested whether the addition of ALI to the GRACE risk score provides an additional benefit in the discriminatory power of GRACE in determining the one-year MACCEs and all-cause mortality. We observed that adding ALI to the GRACE risk score significantly improved the discrimination ability in determining the MACCEs compared to the GRACE risk score and ALI alone, but this improvement was not observed for all-cause mortality (Figure 4).

**Figure 5:**
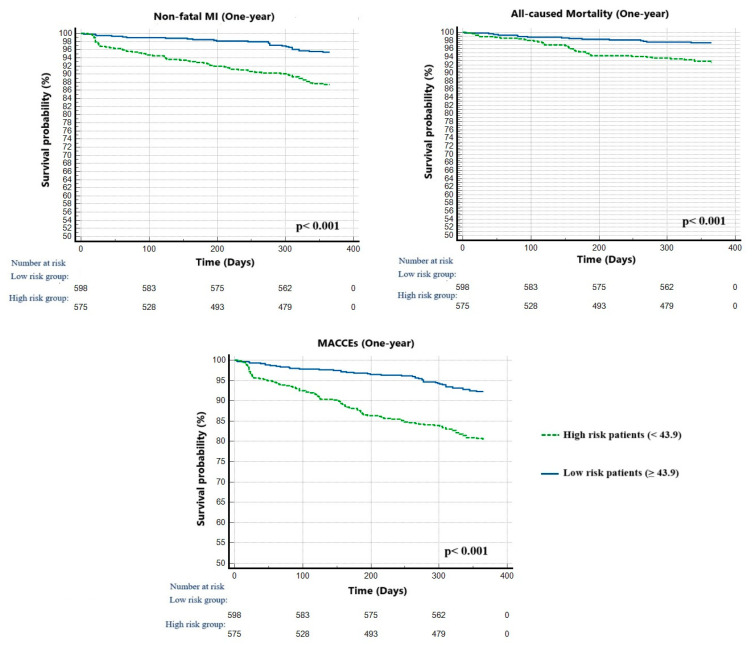
The Kaplan–Meier plots of survival curves of patients with high- (blue line) and low- (green line) ALI categories. Abbreviations: ALI, advanced lung cancer inflammation index; MACCEs, major adverse cardiovascular and cerebrovascular events.

Furthermore, Kaplan–Meier curves represented that high-risk patients with ALI lower than 43.9 had significantly adverse events compared to the low-risk group during the follow-up period (*p* = 0.001) (Figure 5).

## 5. Discussion

The main findings of this retrospective study were as follows: (i) The ALI was an independent associate in determining one-year MACCEs in NSTEMI patients undergoing PCI. (ii) The discriminatory power of ALI in determining the development of MACCEs was statistically better than albumin levels and other inflammatory indices including NLR, CAR and UAR. Furthermore, it had better discrimination ability than parameters other than NLR in terms of all-cause mortality. (iii) An ALI lower than 43.9 related to one-year MACCEs with a 72% sensitivity and a 53% specificity. (iv) Individuals with low ALI had a 2.3-fold increased risk and a significantly worse prognosis. (v) The addition of ALI to the GRACE risk score significantly improved the predictive performance and discriminatory ability of the GRACE risk score alone in determining the MACCEs.

Inflammation plays an important role in the progression and destabilization of atherosclerosis via various mediators and is responsible for initiating ACS by leading to vascular inflammation, changes in hemostasis, plaque rupture and subsequent acute coronary thrombosis [7,8]. The degree of inflammatory activity, increase in vasomotor tone and plaque activation have been proposed as the primary mechanisms associated with clinical instability in the setting of NSTEMI [25]. Furthermore, recent studies focusing on the prognostic role of inflammation have suggested that inflammatory markers are promising for risk stratification and directing therapeutic approaches such as colchicine, which has been proven to improve patient prognosis and overall outcomes in the context of acute coronary events [8,26]. Moreover, there are also data that inflammatory indices can increase the prognostic accuracy of current risk scores mainly derived from clinical, electrocardiographic and angiographic features [25]. Given the link between inflammation and ACS, efforts increasingly continue to identify inflammatory markers associated with prognosis and poor outcome in the setting of MI. One such marker, NLR, has been tested and found to be associated with both short- and long-term mortality in individuals with ACS treated with PCI [27]. CAR, another inflammatory-based marker, has also been investigated in several studies in ACS patients in terms of its relationship with disease severity and poor outcome. Kalyoncuoğlu et al. observed that there was a significant relationship between admission CAR levels and CAD severity in NSTEMI patients and it even showed a better predictive performance than NLR [9]. Additionally, Menekse et al. recently reported that increased CAR levels were associated with in-hospital mortality in NSTEMI patients [10]. Furthermore, in several studies conducted in NSTEMI and STEMI populations, UAR, another inflammatory-based marker, was found to be associated with severe CAD, poor collateral circulation and increased long-term mortality [11,28,29].

Malnutrition is another critical condition that is known to be associated with adverse cardiovascular outcomes such as inflammation in ACS patients and therefore needs to be taken into consideration [8,12,13]. Despite malnutrition being suggested as a correctable risk factor, just like inflammation, and that it may contribute to improving prognosis in ACS patients [8], there is not yet full consensus on their routine clinical use in predicting outcomes due to lack of data [12,13]. Newly identified ALI combines both inflammation and nutritional status represented by NLR and BMI/serum albumin, respectively, and has been investigated for its prognostic significance in several cardiovascular clinical conditions [8,15,16,17]. Chen et al. found that ALI is associated with all-cause and cardiovascular mortality and even left ventricular reverse remodeling in heart failure patients [15]. Additionally, Wang et al. showed that ALI independently associated all-cause mortality and severe heart failure, requiring rehospitalization, in patients with ACS undergoing PCI in their study [16]. The correlation of ALI for long-term major adverse cardiac events in elderly patients with ACS was demonstrated by Zhao et al. [17]. Finally, Trimarchi et al. reported that ALI was an independent prognostic factor for all-cause mortality in STEMI patients treated by PCI and individuals with low ALI experienced a 2.3-fold higher risk of death. They also observed that ALI exhibited greater discriminatory performance than NLR [8]. Despite the relationship between ALI and CAD severity shown in NSTEMI patients [17], the prognostic role of ALI in the NSTEMI population has not been investigated yet, and our study is the first one to focus specifically on the NSTEMI population. The results of our study are compatible with literature data revealing the prognostic importance of ALI. We also observed that ALI had better discriminative ability than NLR and that individuals with low ALI values had a 2.3 times higher risk for the development of MACCEs, similar to previous studies [8,17]. On the other hand, ALI was not superior to NLR in terms of all-cause mortality. When we performed DeLong test analysis separately for nonfatal MI and all-cause mortality, we observed that ALI has a more statistically superior discriminative performance than NLR (AUC = 0.643 vs. AUC = 0.609, *p* = 0.008, respectively) in terms of the development of nonfatal MI, which is a possible reason for this situation. Therefore, this finding suggests that ALI is superior to other inflammatory parameters in terms of future cumulative cardiovascular events rather than just the development of death. In addition, compared with CAR, UAR and albumin, two inflammatory and one malnutrition parameter, respectively, this study revealed better discriminatory power of ALI than these parameters. This finding may be partially attributable to the phenomenon of “the obesity paradox”. Although conflicting results have been obtained and the existence of this protective effect has been questioned, previous studies suggested better short- and medium-term prognosis in overweight ACS patients [30,31,32]. Indeed, Camprubi and colleagues revealed that patients without MACCEs tended to have higher BMI values, reflecting an inverse relationship between BMI and in-hospital mortality for ACS patients [30].

One point to consider is that, compared to previous studies, the ALI value that best discriminates MACCEs in our study was 43.9, and this value appears slightly higher. We think that the possible main reason for this difference is that the studied populations have different BMI, albumin and NLR values and therefore there is inherent variability in ALI values due to the calculation method. In fact, in the study conducted by Zao et al., the patients had lower BMI values and higher NLR values than our study population [17]. In addition, a similar situation was observed in another study conducted by Gong et al. in patients with AMI complicated with cardiogenic shock [16]. In another study by Trimarchi et al., the BMI values of the study population were similar to our study population, but they had lower albumin levels and higher NLR values [8]. Considering the results of these studies mentioned above cumulatively, the fact that our population has higher BMI and albumin values and lower NLR values explains why we have a higher cut-off value. Although it is obvious that more comprehensive prospective studies are needed to reveal a more precise cut-off value, we think that the results of our study are important in terms of revealing the prognostic role of ALI in the NSTEMI population and shedding light on further research on this subject.

In summary, ALI is a composite index derived from BMI, albumin and NLR, which are anthropometric-, nutritional- and inflammation-related indicators, respectively. Therefore, the superiority of ALI over other indexes may be linked to the comprehensive assessment of both inflammatory and nutritional status and providing an assessment of the patient’s condition in a more holistic way. In addition, hepatic T1 mapping, as a recently identified imaging biomarker, has been found to be useful for prognostic stratification after acute myocardial infarction in STEMI patients. Therefore, the ALI index combined with an imaging modality with prognostic significance, such as hepatic T1 mapping, may be more valuable as a prognostic parameter in NSTEMI patients [33]. Thus, ALI could be beneficial for improving clinical outcomes by identifying high-risk patients and allowing for the implementation of data-directed treatment strategies.

Considering the other predictors, consistent with literature data, higher baseline troponin levels, decreased eGFR, higher SxSI, decreased LVEF, incomplete revascularization and diabetes were independently related to the development of MACCEs in our study [8,12,34,35]. On the other hand, although patients who developed MACCEs had a higher GRACE risk score (112.8 ± 27.9 vs. 98.0 ± 26.4, *p* < 0.001), GRACE risk score was not found to be an independent predictor of MACCEs in the current study, consistent with the results of several previous studies [36,37]. GRACE risk score, a traditional prognostic score, was originally developed to estimate the risk of death in hospitals, and the clinical endpoint only takes into account all-cause mortality, excluding other cardiovascular events such as nonfatal myocardial infarction and coronary revascularization. This is likely to be a possible reason for our finding. Furthermore, similar to the study by Zao et al., the current study revealed that the combination of ALI and GRACE scores had strong relation and discriminatory power compared to using ALI and GRACE scores alone [17]. Therefore, the addition of ALI to the GRACE score may help in a comprehensive assessment of the patient’s prognosis and the implementation of more effective preventive measures to reduce the adverse events.

## 6. Limitations

Our study has some limitations that should be considered. First, this study is a relatively small-sized, retrospective study based only on data accessed through the hospital information system and reflects the experience of a single center. Due to its retrospective nature, it was able to establish associations but could not establish causality. The relatively small sample size may make this study underpowered to detect the value of ALI in predicting one-year MACCEs. Second, the study focused only on admission ALI levels and ignored time-varying changes, which may provide more valuable information for understanding underlying mechanisms. Third, despite our efforts to comprehensively adjust for many important covariates, other unknown factors (e.g., treatment adherence issues) may be present and introduce bias. Fourth, the presence of confounding clinical conditions that may be associated with the development of malnutrition, such as undetected cancer, psychiatric disorders and hypothyroidism, have also not been investigated. Fifth, patients with CABG were not included in the study because SxSI could not be calculated. Moreover, only NSTEMI patients who underwent PCI were included in this study and do not represent the entire NSTEMI population, including patients for whom medical treatment decisions or CABG decisions were made. Sixth, the findings obtained in the current study are exploratory and should be considered as hypothesis-generating. Therefore, further well-designed, prospective multi-center studies are needed to confirm and expand the results of our study.

## 7. Conclusions

In conclusion, our study highlights the association of ALI with the development of one-year MACCEs in NSTEMI patients undergoing PCI. The findings also underline that ALI has superior discriminatory ability, strong relationships and more pronounced benefit for risk stratification in this patient group compared to previously defined inflammatory parameters such as NLR, CAR and UAR. ALI is a simple, easily accessible and cost-effective marker that may be useful by allowing for the comprehensive assessment of a patient’s overall condition. Finally, the results of our study not only reveal the prognostic role of ALI but also highlight the need for further research to elucidate the impact of ALI on outcomes in NSTEMI patients undergoing revascularization treatment with PCI.

## Figures and Tables

**Table 1:** Demographic and clinical characteristics of study population.

Variables	All Population (n = 1173)	Low ALI (<43.9) (n = 575; 49.0%)	High ALI (≥43.9) (n = 598; 51.0%)	*p*
Female gender, n %	296 (25.2)	149 (25.9)	147 (24.6)	0.600
Age	61.9 ± 12.5	62.1 ± 12.7	61.6 ± 12.4	0.491
BMI (kg/m^2^)	27.7 ± 3.4	26.9 ± 3.4	28.4 ± 3.3	<0.001
Hypertension, n (%)	674 (57.5)	324 (56.3)	350 (58.5)	0.450
Diabetes, n (%)	417 (35.5)	207 (36.0)	210 (35.1)	0.752
Hyperlipidemia, n (%)	548 (46.7)	268 (46.6)	280 (46.8)	0.941
Family history, n (%)	411 (35.0)	214 (37.2)	197 (32.9)	0.125
Smoking, n (%)	522 (44.5)	269 (46.8)	253 (42.3)	0.123
CAD history, n (%)	500 (42.6)	254 (44.2)	246 (41.1)	0.293
MI history, n (%)	362 (30.9)	189 (32.9)	173 (28.9)	0.144
PCI history, n (%)	376 (32.1)	192 (33.4)	184 (30.8)	0.336
PAD history, n (%)	33 (2.8)	19 (3.3)	14 (2.3)	0.319
CRF, n (%)	132 (11.3)	70 (12.2)	62 (10.4)	0.328
CRF, dialysis, n (%)	11 (0.9)	5 (0.9)	6 (1.0)	0.812
Killip II-IV, n (%)	105 (9.0)	57 (9.9)	48 (8.0)	0.258
GRACE score	100.0 ± 27.0	101.9 ± 28.2	98.0 ± 25.7	0.014
LVEF (%)	51.3 ± 9.9	50.3 ± 10.4	52.2 ± 9.3	0.002
Hemodynamic instability, n (%)	35 (3.0)	25 (4.3)	10 (1.7)	0.007
Medications, n (%)				
Acetylsalicyclic acid	424 (36.1)	210 (36.5)	214 (35.8)	0.793
Clopidogrel	137 (11.7)	76 (13.2)	61 (10.2)	0.108
Oral anticoagulants	57 (4.9)	31 (5.4)	26 (4.4)	0.410
Beta-Blockers	348 (29.7)	177 (30.8)	171 (28.6)	0.412
RAS Blockers	549 (46.8)	255 (44.3)	294 (49.2)	0.098
Calcium-channel blockers	424 (36.1)	201 (35.0)	223 (37.3)	0.405
Statin	264 (22.5)	129 (22.4)	135 (22.6)	0.954
Antianginals	115 (9.8)	59 (10.3)	56 (9.4)	0.606
Syntax Score I	20.1 ± 6.0	20.7 ± 6.3	19.6 ± 5.7	0.001
‡ Complete revascularization	564 (48.1)	271 (47.1)	293 (49.0)	0.522
* TIMI < 3	102 (8.7)	47 (8.2)	55 (9.2)	0.534
* Stent thrombosis	14 (1.2)	9(1.6)	5 (0.8)	0.250
* Require TVR, n (%)	39 (3.3)	24(4.2)	15 (2.5)	0.112
Nonfatal MI, n (%)	96 (8.2)	69 (12.0)	27 (4.5)	<0.001
Nonfatal stroke, n (%)	7 (0.6)	3 (0.5)	4 (0.7)	0.744
Death, all-cause, n (%)	55 (4.7)	40 (7.0)	15 (2.5)	<0.001
MACCEs, n (%)	158 (13.5)	112 (19.5)	46 (7.7)	<0.001

‡ Complete revascularization in hospitalization period. * Not a component of MACCEs. Abbreviations: BMI, body mass index; CAD, coronary artery disease; CRF, chronic renal failure; GRACE, Global Registry of Acute Coronary Events; LVEF, left ventricular ejection fraction; MI, myocardial infarction; PAD, peripheral artery disease; PCI, percutaneous coronary intervention; RAS, renin-angiotensin system; TIMI, Thrombolysis in Myocardial Infarction; TVR, target vessel revascularization.

**Table 2:** Laboratory parameters of study population.

Variables	All Population (n = 1173)	Low ALI (<43.9) (n = 575; 49.0%)	High ALI (≥43.9) (n = 598; 51.0%)	*p*
Glucose, mg/dL	114.8 ± 32.5	115.5 ± 33.2	114.1 ± 31.8	0.457
eGFR, mL/min/1.73 m^2^	80.8 ± 23.9	80.0 ± 24.2	81.6 ± 23.5	0.240
Serum uric acid, mg/dL	5.52 ± 1.8	5.62 ± 1.8	5.42 ± 1.7	0.053
Albumin, g/dL	3.88 ± 0.48	3.75 ±0.47	4.0 ± 0.44	<0.001
CRP, mg/dL, IQR	5.36 [3.45–8.23]	5.45 [3.50–8.60]	5.23 [3.30–8.00]	0.131
Baseline Troponin I, ng/mL	0.06 [0.02–0.21]	0.060 [0.021–0.23]	0.058 [0.020–0.19]	0.011
TC, mg/dL	193.2 ± 43.5	193.2 ± 41.2	193.3 ± 45.7	0.968
LDL-C, mg/dL	117.1 ± 37.6	117.0 ± 35.6	117.3 ± 39.5	0.893
HDL-C, mg/dL	39.2 ± 10.3	39.5 ± 10.9	38.9 ± 9.6	0.348
Triglyceride, mg/dL	122.0 [99.0–167.0]	121.0 [100.0–167.0]	123.0 [99.0–169.0]	0.867
Hemoglobin, g/dL	13.7 ± 1.9	13.6 ± 1.9	13.8 ± 1.8	0.065
WBC, 10^9^/L	8.56 ± 2.2	8.96 ± 2.6	8.18 ± 1.8	<0.001
Neutrophil, 10^9^/L	5.29 ± 1.9	6.07 ± 2.1	4.54 ± 1.2	<0.001
Lymphocyte, 10^9^/L	2.06 [1.58–2.58]	1.69 [1.28–2.15]	2.43 [1.98–2.95]	<0.001
Platelet, 10^9^/L	256.7 ± 76.4	255.6 ± 77.9	253.1 ± 72.0	0.565
NLR	2.40 [1.85–3.30]	3.30 [2.74–4.34]	1.87 [1.54–2.23]	<0.001
ALI, kg × g/m^2^ × dL	43.7 [31.1–58.5]	30.8 [23.0–37.2]	57.9 [50.3–73.5]	<0.001
CAR, mg/g	1.42 [0.88–2.17]	1.51 [0.92–2.35]	1.30 [0.84–2.0]	<0.001
UAR, mg/g	1.45 ± 0.5	1.54 ± 0.6	1.37 ± 0.5	<0.001

Abbreviations: ALI, advanced lung cancer inflammation index; CAR, C-reactive protein to albumin ratio; CRP, C-reactive protein; eGFR, estimated glomerular filtration rate; HDL-C, high-density lipoprotein cholesterol; LDL-C, low-density lipoprotein cholesterol; NLR, neutrophil to lymphocyte ratio; TC, total cholesterol; UAR, uric acid to albumin ratio; WBC, white blood count.

**Table 3:** Parameters associated with MACCEs in univariate Cox regression analysis.

Clinical Variables			Laboratory Variables		
	HR (95% CI)	*p*		HR (95% CI)	*p*
Age	1.030 (1.017–1.044)	<0.001	eGFR	0.978 (0.972–0.983)	<0.001
Gender	1.689 (1.219–2.341)	0.002	Albumin	0.531 (0.396–0.711)	<0.001
Diabetes	2.125 (1.555–2.904)	0.001	Uric acid	1.104 (1.014–1.202)	0.022
CAD	1.599 (1.170–2.185)	0.003	Troponin	1.423 (1.333–1.519)	<0.001
GRACE	1.020 (1.014–1.026)	<0.001	Hemoglobin	0.796 (0.739–0.857)	<0.001
LVEF	0.951 (0.939–0.964)	<0.001	NLR	1.170 (1.109–1.234)	<0.001
SxSI	1.061 (1.039–1.083)	<0.001	ALI	0.971 (0.962–0.979)	<0.001
TIMI < 3	1.822 (1.162–2.859)	0.009	CAR	1.238 (1.071–1.430)	0.004
CR	0.974 (0.264–0.532)	<0.001	UAR	1.644 (1.272–2.125)	<0.001

Abbreviations: ALI, advanced lung cancer inflammation index; CAD, coronary artery disease; CAR, C-reactive protein to albumin ratio; CR, complete revascularization; eGFR, estimated glomerular filtration rate; GRACE, Global Registry of Acute Coronary Events; LVEF, left ventricular ejection fraction; NLR, neutrophil to lymphocyte ratio; SxSI, anatomical syntax score I; TIMI, Thrombolysis in Myocardial Infarction; UAR, uric acid to albumin ratio.

**Table 4:** Factors that were found to be independently associated with MACCEs in multivariate Cox regression analysis models.

	Model 1 *		Model 2 *		Model 3 *	
Variables	HR (95% CI)	*p*	HR (95% CI)	*p*	HR (95% CI)	*p*
Age	0.983 (0.958–1.008)	0.174	0.986 (0.961–1.012)	0.278	0.986 (0.961–1.012)	0.291
Gender	1.274 (0.880–1.845)	0.200	1.282 (0.882–1.864)	0.192	1.325 (0.913–1.924)	0.139
Diabetes	1.470 (1.047–2.063)	0.026	1.475 (1.052–2.069)	0.024	1.447 (1.032–2.030)	0.032
CAD	1.257 (0.904–1.748)	0.174	1.222 (0.879–1.698)	0.233	1.220 (0.878–1.695)	0.237
GRACE score	1.009 (0.997–1.022)	0.141	1.008 (0.995–1.020)	0.238	1.007 (0.995–1.020)	0.251
LVEF	0.981 (0.967–0.996)	0.014	0.984 (0.969–0.999)	0.035	0.983 (0.968–0.998)	0.027
SxSI	1.050 (1.027–1.072)	<0.001	1.058 (1.036–1.080)	<0.001	1.056 (1.034–1.079)	<0.001
TIMI < 3	1.424 (0.895–2.265)	0.135	1.322 (0.831–2.102)	0.239	1.393 (0.876–2.214)	0.161
CR	0.514 (0.356–0.742)	<0.001	0.509 (0.352–0.736)	<0.001	0.510 (0.353–0.737)	<0.001
eGFR	0.987 (0.979–0.995)	0.001	0.988 (0.980–0.996)	0.003	0.988 (0.980–0.997)	0.005
Uric acid	1.047 (0.962–1.140)	0.290	1.051 (0.967–1.142)	0.246	-	-
Troponin	1.358 (1.257–1.468)	<0.001	1.373 (1.270–1.486)	<0.001	1.370 (1.266–1.481)	<0.001
Hemoglobin	0.992 (0.902–1.090)	0.865	0.984 (0.895–1.083)	0.748	0.988 (0.897–1.088)	0.800
NLR	-	-	1.122 (1.059–1.188)	<0.001	1.127 (1.065–1.193)	<0.001
ALI	0.974 (0.965–0.982)	<0.001	-	-	-	-
CAR	-	-	1.232 (1.063–1.428)	0.006	-	-
UAR	-	-	-	-	1.404 (1.092–1.806)	0.008

* It refers to multivariate Cox regression analysis models using the “ENTER” method based on ALI, CAR and UAR, respectively. Abbreviations: ALI, advanced lung cancer inflammation index; CAD, coronary artery disease; CAR, C-reactive protein to albumin ratio; CR, complete revascularization; eGFR, estimated glomerular filtration rate; GRACE, Global Registry of Acute Coronary Events; LVEF, left ventricular ejection fraction; NLR, neutrophil to lymphocyte ratio; SxSI, anatomical syntax score I; TIMI, Thrombolysis in Myocardial Infarction; UAR, uric acid to albumin ratio.

## Data Availability

The original contributions presented in the study are included in the article; further inquiries can be directed to the corresponding authors.
